# Characterization of QTLs for diameter in panicle neck and substitution mapping of *qDPN5/qVBN5.2* and *qVBN6* in rice (*Oryza sativa* L.)

**DOI:** 10.1270/jsbbs.23076

**Published:** 2024-08-14

**Authors:** Ha Thi Le Nguyen, Ami Yoshiura, Shao-Hui Zheng, Daisuke Fujita

**Affiliations:** 1 The United Graduate School of Agricultural Sciences, Kagoshima University, 1-21-24 Korimoto, Kagoshima 890-8580, Japan; 2 Forest Science Institute of South Vietnam, 1 Pham Van Hai, Tan Binh District, Ho Chi Minh City, Viet Nam; 3 Faculty of Agriculture, Saga University, 1 Honjo-machi, Saga 840-8502, Japan

**Keywords:** CSSL, panicle neck diameter, pyramided line

## Abstract

The vascular bundle system in the panicle neck of rice (*Oryza sativa* L.) connects the culm to the panicle and transports assimilates. The number of vascular bundles in the panicle neck (VBN) is correlated with the diameter of the panicle neck (DPN), but there are few reported QTLs for DPN. We conducted quantitative trait locus (QTL) analysis using recombinant inbred lines (RILs) derived from a cross between ‘Asominori’ and ‘IR24’ and detected three QTLs—*qDPN5*, *qDPN6*, and *qDPN11*—on chromosomes 5, 6, and 11. The *qDPN5*, *qDPN6*, and *qDPN11* were in the same position as the QTLs for VBN reported in previous studies. Within the RILs, there was a significant positive correlation between DPN and VBN. In segregating populations, each QTL had a distinct effect on both values. Analysis of chromosome segment substitution lines showed that *qDPN5* and *qDPN11* affected DPN and *qDPN6* affected VBN. Through substitution mapping, we narrowed down the region of *qDPN5* and *qVBN5.2* to 960 kbp between KNJ8 Indel385 and RM18926, and the region of *qVBN6* to 750 kbp between C5 Indel5756 and KNJ8 Indel493. Due to the weak effect of *qDPN6* in the ‘IR24’ genetic background, the location of *qDPN6* could not be determined.

## Introduction

Vascular bundles in rice (*Oryza sativa* L.) play an important role in transporting photosynthates, nutrients, and water throughout the plant (Lucas *et al.* 2013). Vascular bundles in the panicle neck of rice are critical for the transport of photosynthates from source to sink (Cui *et al.* 2003, Fukuyama *et al.* 1999). To improve rice production, it is essential to increase both source and sink sizes, as well as the capacity for translocation. When the vascular bundle number in the panicle neck (VBN) is limited, assimilate transport and grain filling are restricted (Peng *et al.* 1999, Xu *et al.* 2005). The diameter of the panicle neck (DPN), which is highly correlated with VBN, strongly affects source-to-sink matter transfer (Lee *et al.* 1992, Xu *et al.* 2005). In addition, DPN is positively correlated with sink size in the form of primary and secondary branch numbers and spikelet number (Lee *et al.* 1992, Liao *et al.* 2021, Liu *et al.* 2008, Xu *et al.* 2005).

Subspecies *indica* rice generally has a higher VBN than *japonica* rice (Fukuyama and Takayama 1995, Fukuyama *et al.* 1999, Liu *et al.* 2016, Zhai *et al.* 2018), with a varying ratio of VBN to primary branch number (Fukuyama and Takayama 1995, Fukuyama *et al.* 1999). VBN is influenced by both genetic and environmental factors such as nitrogen availability and plant density. Through the use of segregating populations such as recombinant inbred lines (RILs) and doubled haploid lines (DHs), genetic factors for VBN have been detected, and genome-wide association studies (GWAS) have identified associated regions (Bai *et al.* 2012, Cui *et al.* 2003, Liao *et al.* 2021, Nguyen *et al.* 2023, Sasahara *et al.* 1999, Zhai *et al.* 2018, Zhang *et al.* 2002). Some of the genes responsible for VBN have been cloned and isolated including *ABERRANT PANICLE* *ORGANIZATION 1* (*APO1*) on chromosome (Chr.) 6 (Terao *et al.* 2010), *NARROW LEAF 1* (*NAL1*) on Chr. 4 (Fujita *et al.* 2013, Qi *et al.* 2008), and *LVB9/DENSE AND ERECT PANICLE 1* (*DEP1*) on Chr. 9 (Fei *et al.* 2019).

QTLs for DPN have been detected on Chrs. 4, 6, and 11 in RILs derived from ‘Zhenshan 97B’ and ‘IRAT109’ upland rice (Liu *et al.* 2008). Liao *et al.* (2021) detected QTLs for DPN on Chrs. 1–7, 11, and 12 by GWAS of introgression lines derived from combinations of ‘Zhenshan 97’, ‘Nipponbare’, ‘Huanghuazhan’, ‘Uz-Rosz 275’, and ‘Celiaj’. Jeon *et al.* (2018) narrowed down *FID2* for DPN on Chr. 2 in a segregating population derived from an interspecific hybrid between *japonica* ‘Hwaseongbyeo’ and *Oryza grandiglumis*.

We identified three stable QTLs for VBN on Chrs. 5, 6, and 11 in RILs derived from a cross between *japonica* ‘Asominori’ and *indica* ‘IR24’ (Nguyen *et al.* 2023) and evaluated their effects alone and in pairs using chromosome segment substitution lines (CSSLs) and pyramided lines (PYLs) with an ‘IR24’ genetic background. We could not identify their precise locations or clarify the correlation between VBN and DPN. Here, we had three objectives: (1) to reveal genetic factors that control DPN; (2) to reveal the relationship between VBN and DPN; and (3) to delimit the regions of the QTLs on Chrs. 5 and 6. We conducted QTL analysis for DPN using RILs and characterized QTL effects using CSSLs and PYLs; revealed the relationship between VBN and DPN using RILs and an F_2_ population; and delimited the region of QTLs for VBN and DPN by substitution mapping.

## Materials and Methods

### Plant materials

To conduct QTL analysis for DPN, we used 71 RILs derived via single-seed descent from a cross between *japonica* ‘Asominori’ and *indica* ‘IR24’ (Tsunematsu *et al.* 1996). To evaluate the effect of the three QTLs in 2020 and 2021, we used three CSSLs—IAS14 (*qDPN11*), IAS30 (*qDPN5*) and IAS39 (*qDPN6*)—carrying target chromosomal segments of ‘Asominori’ in the ‘IR24’ genetic background (Kubo *et al.* 2002). Since the locations of these QTLs for DPN coincided with those of QTLs for VBN, we used PYLs developed by Nguyen *et al.* (2023) to evaluate DPN for each combination. For evaluating the effects of DPN on PYL with three QTLs, F_1_ derived from the crosses between PYLs with two QTLs were generated (Nguyen *et al.* 2023) and was self-pollinated to produce F_2_. Using SSR markers (Supplemental Table 1), one plant with the ‘Asominori’ homozygous allele at three QTLs was selected among the 96 F_2_ plants. In 2022, the F_3_ line from selected F_2_ plant was assessed for VBN and DPN. To evaluate the effect of ‘IR24’ alleles of QTLs with ‘Asominori’ genetic background, we used three CSSLs—AIS38 (carrying *qDPN5*), AIS49 (carrying *qDPN6*), and AIS76 (carrying *qDPN11*)—carrying target chromosomal segments of ‘IR24’ in the ‘Asominori’ genetic background (Kubo *et al.* 2002).

### Materials for substitution mapping

We used two F_2_ populations (168 plants each) derived from IAS30/IR24 and IAS39/IR24 to confirm QTLs for DPN (Supplemental Fig. 1). To conduct substitution mapping, we selected plants with recombination around *qDPN5* and *qDPN6* from each F_2_ population and self-pollinated them to produce F_3_ lines. For substitution mapping of *qDPN5*, we analyzed 54 F_3_ lines using 10 additional markers and selected 19 plants with homozygous recombination around *qDPN5*. Following self-pollination, we developed 19 F_4_ lines and measured VBN and DPN. Finally, we measured VBN of 19 F_5_ lines to delimit the location of *qDPN5*. For substitution mapping of *qDPN6*, we analyzed 44 F_3_ lines using 9 additional markers, and selected 40 plants with homozygous recombination around *qDPN6*. Following self-pollination, we developed 40 F_4_ lines and measured VBN and DPN. We measured VBN of 12 F_5_ lines to delimit the location of *qDPN6*.

### Evaluation of VBN and diameter of the panicle neck

The plants were grown in a paddy field at Saga University (33°14ʹ32ʺN 130°17ʹ24ʺE) from May to October. Seedlings were transplanted 28 days after germination at one plant per hill, at 20 cm between hills and 25 cm between rows. Inorganic fertilizer was applied at 40 kg/ha N, 17.5 kg/ha P, and 33 kg/ha K. Each F_3_, F_4_, and F_5_ line consisted of 16–24 plants in 3 rows. Five plants were harvested from the second or third rows (excluding plants at the end of each row) 2 weeks after flowering of the tallest panicle. Fresh peduncles were cut less than 1 mm about 1 cm below the panicle base node and observed VBN and DPN under a microscope. Microscope was used to capture images of the oval cross-section of the panicle neck node. The maximum width of the oval cross-section was measured as DPN using Image J (National Institutes of Health, Bethesda, MD, USA) software. For each line, 5–10 samples of VBN and DPN were recorded.

### DNA extraction and genotyping

Fresh leaf (2–4 cm) was freeze-dried for 48 h, and DNA was extracted by the potassium acetate method (Dellaporta *et al.* 1983). GoTaq Master Mix (Promega) was used for PCR amplification in 35 cycles of 30 s at 96°C, 30 s at 55°C, and 30 s at 72°C, followed by a final extension at 25°C for 1 min. The PCR products were separated by electrophoresis in 4% agarose gel containing 0.5 μg/mL ethidium bromide in 0.5 TBE buffer at 200 V for 1–2 h. Using genotyping data from RFLP markers (Tsunematsu *et al.* 1996), we conducted QTL analysis for DPN. QTLs were confirmed using the same markers used for identifying the VBN QTL in the F_2_ plants of IAS30/IR24 and IAS39/IR24 (Nguyen *et al.* 2023). For substitution mapping in F_3_ recombinant lines, we used polymorphic SSR and indel markers (McCouch *et al.* 2002, Yonemaru *et al.* 2015). The F_3_ plants derived from 54 F_2_ plants recombinant around *qDPN5* between RM7081 and RM7446 were genotyped using 10 markers. Similarly, the F_3_ plants derived from 44 F_2_ plants recombinant around *qDPN6* near RM6395 and RM20546 were genotyped using nine markers (Supplemental Table 2).

### QTL analysis

Composite interval mapping (CIM) was performed in Windows QTL Cartographer v. 2.5 software (Wang *et al.* 2012). The significance threshold was determined by 1000 permutation tests, with a logarithm of odds (LOD) score of 3.0 at *P* < 0.05.

### Statistical analysis

One-way ANOVA was conducted to compare the mean values of VBN and DPN of homozygous recombinants. Dunnett’s test was used to analyze the phenotypic differences between homozygous recombinant lines and ‘IR24’. The Tukey–Kramer comparison test was conducted to compare differences in DPN among lines.

## Results

### Identification of QTLs for diameter of panicle neck in RILs

DPN was 1.70 mm in ‘Asominori’ and 2.55 mm in ‘IR24’ (Supplemental Fig. 2). The frequency distribution of DPN in RILs ranged from 1.7 to 2.7 mm and was continuous, indicating that multiple genetic factors control DPN. QTL analysis detected three QTLs for DPN: *qDPN5* on Chr. 5, *qDPN6* on Chr. 6, and *qDPN11* on Chr. 11 (Table 1). The ‘Asominori’ alleles at those QTLs decreased DPN, with proportion of variance explained (PVE) of 17.3% in *qDPN5*, 38.1% in *qDPN6*, and 11.7% in *qDPN11* (total = 67.1%).

### Validation of single-QTL effect on DPN in ‘IR24’ genetic background

To assess the effect of single QTLs for DPN, we measured the DPN of CSSLs carrying the ‘Asominori’ alleles, IAS30 (*qDPN5*), IAS39 (*qDPN6*) and IAS14 (*qDPN11*) in 2 years. In 2020, the ‘Asominori’ alleles significantly decreased DPN by 0.41 mm in IAS14, by 0.46 mm in IAS30 and by 0.29 mm in IAS39, relative to ‘IR24’ (2.74 mm, Table 2). In 2021, they significantly decreased DPN by 0.38 mm in IAS14, by 0.56 mm in IAS30 and by 0.19 mm in IAS39, relative to ‘IR24’ (2.57 mm, Table 2). The effects were consistent between years. Additionally, the VBN of IAS30, IAS39, and IAS14 was characterized and ‘Asominori’ alleles significantly decreased VBN by 5.2 in IAS14, by 6.4 in IAS39, and by 1.6 in IAS30, relative to ‘IR24’ (22.2, Table 2).

### Evaluation of pyramiding effects of QTLs for DPN in ‘IR24’ genetic background

To assess the interactions of QTLs associated with DPN, we used PYLs with multiple QTLs. Table 3 indicates that DPN of PYL1 (*qDPN5* + *qDPN11*) was 2.02 mm in 2020 and 1.89 mm in 2021, similar to that of parental lines IAS30 (*qDPN5*) and IAS14 (*qDPN11*), and ‘Asominori’ but significantly smaller than that of ‘IR24’ in both years. DPN of PYL2 (*qDPN6* + *qDPN11*) was 2.26 mm in 2020 and 2.04 mm in 2021, similar to that of IAS14 but significantly smaller than that of ‘IR24’ in both years, and similar to that of IAS39 (*qDPN6*) in 2020 but significantly smaller in 2021. DPN of PYL3 (*qDPN5* + *qDPN6*) was 2.06 mm in 2020 and 1.98 mm in 2021, similar to that of IAS30 and ‘Asominori’ but significantly smaller than those of IAS39 and ‘IR24’ in both years, and significantly larger than that of ‘Asominori’ in 2020 but similar in 2021. In 2022, the DPN tendency in PYL1-2 was consistent with 2020 and 2021, while PYL3 showed the same DPN as the parental line IAS39. In 2022, DPN of PYL4 carrying three QTLs for DPN (*qDPN5+6+11*) was 1.79 mm, which was similar to that of other PYLs carrying *qDPN5+11*, *qPDN6+11*, and *qDPN5+6* (Table 3). DPN of PYL4 was significantly different from IAS39, IAS14, and ‘IR24’. Additionally, VBN of PYL4 (*qDPN5+6+11*) was 11.2, similar to that of PYL2 (*qDPN6+11*) and ‘Asominori’ (Supplemental Fig. 3). The VBN of PYL4 was significantly lower than that of IAS30, IAS39, IAS14, PYL1 (*qDPN5+11*), and PYL3 (*qDPN5+6*).

### Confirmation of QTLs for DPN

To confirm detected QTLs for DPN in RILs, we conducted QTL analysis using populations segregating at a single QTL. The frequency distribution of DPN in the F_2_ population derived from IR24/IAS30 ranged from 1.8 to 3.0 mm (Supplemental Fig. 4A). A single QTL, *qDPN5*, was detected on Chr. 5, with a PVE of 15.9%. The ‘Asominori’ allele decreased DPN by 0.04 mm (Table 4). Similarly, the frequency distribution of DPN in the F_2_ population derived from IR24/IAS39 ranged from 2.0 to 3.1 mm (Supplemental Fig. 4B). A single QTL, *qDPN6*, was detected on Chr. 6, with a PVE of 33.7%. The ‘Asominori’ allele decreased DPN by 0.06 mm (Table 4). These results confirm the presence of these two QTLs for DPN, and the ‘Asominori’ alleles decreased DPN. Based on the nearest markers to *qDPN5*, the DPN of plants with homozygous for ‘Asominori’ was 2.21 mm and the DPN of plants with homozygous for ‘IR24’ was 2.45 mm (Supplemental Fig. 4A). Based on the nearest markers to *qDPN6*, DPN of plants with homozygous for ‘Asominori’ was 2.23 mm and the DPN of plants with homozygous for ‘IR24’ was 2.58 mm (Supplemental Fig. 4B). Since these QTLs overlap with QTLs detected for VBN in our previous study (Nguyen *et al.* 2023), suggesting a relationship between DPN and VBN, we conducted correlation analysis between VBN and DPN in the RILs and F_2_ populations. In the RILs derived from a cross between ‘Asominori’ and ‘IR24’, there was a positive correlation (*r* = 0.55, *P* < 0.001; *y* = 6.04*x* + 3.00; Supplemental Fig. 5). In the F_2_ populations derived from IAS30/IR24 and IAS39/IR24, there were also positive correlations (*r* = 0.44 and 0.6, *P* < 0.001).

### Substitution mapping of *qDPN5* and *qVBN5.2*

**Fig. 1.**
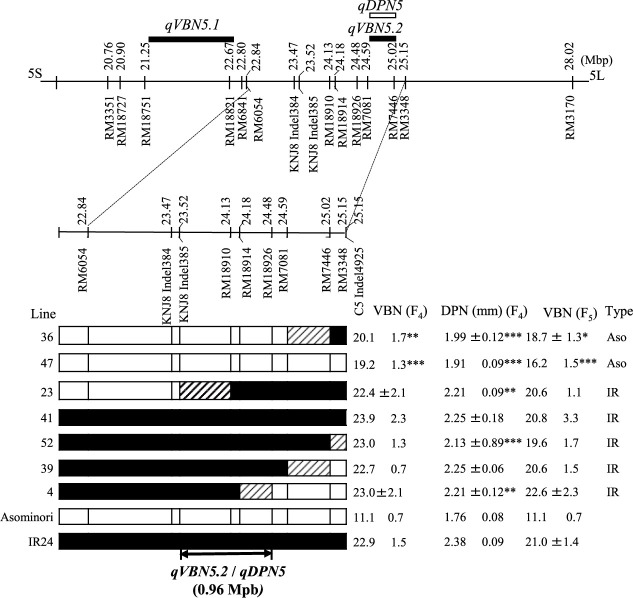
Chromosomal locations of *qVBN5.2* and *qDPN5* by substitution mapping. Boxes indicate genotypes homozygous for 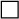 ‘Asominori’ and 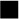 ‘IR24’, or 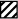 recombinant. ‘IR’ and ‘Aso’ types mean ‘IR24’ and ‘Asominori’. ‘Asominori’ or ‘IR24’ types were classified based on VBN in each line. Asterisks indicate significant differences from ‘IR24’ at *P*-values of *5%, **1%, and ***0.1% by Dunnett’s multiple comparison test.

In our previous study, we identified two QTLs, *qVBN5.1* and *qVBN5.2*, in F_2_ populations derived from IAS30/IR24 (Nguyen *et al.* 2023). *qVBN5.2* was co-located with *qDPN5* between RM7081 and RM7446. To delimit *qDPN5* and *qVBN5.2*, we conducted substitution mapping. VBN of lines carrying the ‘Asominori’ segment varied from 19.2 to 23.9 in F_4_ and from 16.2 to 22.6 in F_5_ (Fig. 1). DPN of F_4_ lines carrying the ‘Asominori’ segment varied consistently with VBN, from 1.91 to 2.25 mm. Among them, VBN of lines 4 and 23 did not differ from that of ‘IR24’ in F_4_ or F_5_, but DPN of these lines was significantly smaller than that of ‘IR24’. The types of ‘IR24’ and ‘Asominori’ of each line in Fig. 1 were classified based on the VBN. On the basis of the recombination between KNJ8 Indel385 and RM18926 in lines 4 and 23, we delimited *qDPN5* and *qVBN5.2* to ~960 kbp between these markers on the ‘Nipponbare’ genome sequence.

### Substitution mapping of *qVBN6* (but not *qDPN6*)

**Fig. 2.**
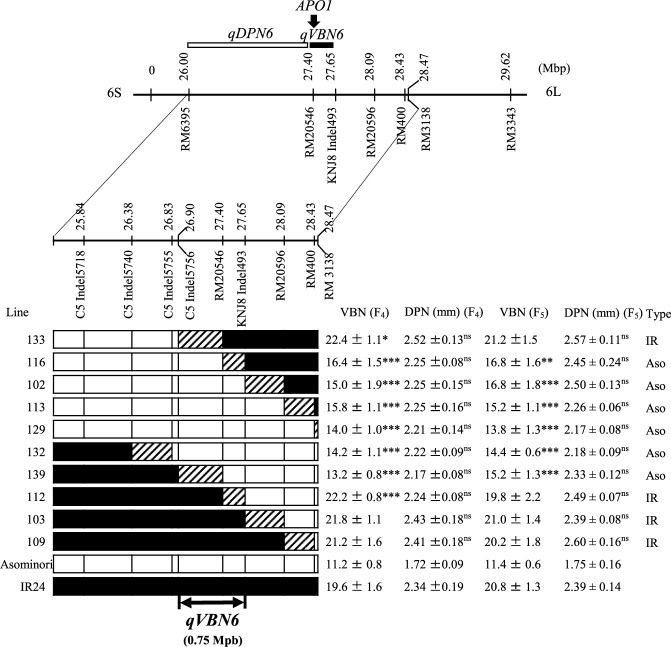
Chromosomal locations of *qVBN6* by substitution mapping. Boxes indicate genotypes homozygous for 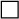 ‘Asominori’ and 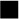 ‘IR24’, or 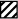 recombinant. ‘IR’ and ‘Aso’ types mean ‘IR24’ and ‘Asominori’. ‘Asominori’ or ‘IR24’ types were classified based on VBN in each line. Asterisks indicate significant differences from ‘IR24’ at *P*-values of *5%, **1%, and ***0.1% by Dunnett’s multiple comparison test; ns, not significant.

In our previous study, we identified *qVBN6* for VBN on Chr. 6 in an F_2_ population derived from IAS39/IR24, near marker RM20546 for *qDPN6*. To delimit *qDPN6* and *qVBN6*, we conducted substitution mapping. VBN of lines carrying the ‘Asominori’ segment varied from 13.2 to 22.4 in F_4_ and from 13.8 to 21.2 in F_5_, but DPN did not differ significantly among these lines (Fig. 2). Among them, VBN of lines 116, 102, 113, 129, 132, and 139 was significantly lower than that of ‘IR24’, whereas that of lines 133, 112, 103, and 109 was similar to that of ‘IR24’ in F_4_ and F_5_. Similarly, ‘IR24’ type and ‘Asominori’ type of each lines in Fig. 2 were classified based on the VBN. On the basis of the recombination between C5 Indel5756 and KNJ8 Indel493 in lines 133, 116, 139, and 112, we delimited *qVBN6* to ~750 kbp between these markers on the ‘Nipponbare’ genome sequence. Although DPN tended to be reduced in lines with low VBN, we could not estimate the location of *qDPN6*.

## Discussion

Several loci for DPN have been identified in segregating populations and by GWAS (Jeon *et al.* 2018, Liao *et al.* 2021, Liu *et al.* 2008). So far, one QTL for DPN, *qFID2*, on Chr. 2, has been identified by substitution mapping (Jeon *et al.* 2018), but its gene function has not been determined. Here, we identified three QTLs for DPN, the locations of which overlap those reported previously: *qDPN5* at the same location as a QTL for DPN at 18.38–29.16 Mbp on Chr. 5, *qDPN6* at the same location as a QTL for DPN at 28.0–31.2 Mbp on Chr. 6, and *qDPN11* near a QTL for DPN at 0.8–2.1 Mbp on Chr. 11 (Liao *et al.* 2021). The three QTLs had a total PVE of 67.1% (Table 1), so other genetic factors might also contribute. These results will help explain the genetic basis of DPN in rice, and could lead to precise mapping and cloning of related loci.

The locations of the QTLs for DPN detected here correspond to known QTLs for VBN: *qVBN5* at ~23.0 Mbp, *qVBN6* at ~28.6 Mbp, and *qVBN11* at ~3.8 Mbp (Nguyen *et al.* 2023). The *qDPN11* was co-located with *qVBN11* at ~2.45 Mpb on Chr. 11 and substitution mapping and characterization of *qDPN11* and *qVBN11* are currently conducting for future publication. *qDPN5* and *qDPN6* were confirmed in the F_2_ population (Supplemental Fig. 4, Table 4). The results of QTL analysis showed that these QTLs exhibited incomplete dominance effects because the dominance values were smaller than the values of additive effect (Table 4). In addition, the frequency distribution of DPN on F_2_ populations based on plants with homozygous for ‘Asominori’ and ‘IR24’ and heterozygous suggests that there might be one QTL controlling DPN in each population (Supplemental Fig. 4). We delimited the regions of *qVBN5.2* and *qDPN5* to ~960 kbp on the ‘Nipponbare’ genome sequence (Fig. 1). There are no reported genes for DPN here, and this locus might have pleiotropic effects on VBN and DPN. *qVBN6* and *qDPN6* were delimited to ~750 kbp, near *APO1* (Os06g0665400) at ~27.46 Mbp on Chr. 6. *APO1* enhances the development of vascular bundles, promoting carbohydrate translocation to the panicle (Terao *et al.* 2010), so *qVBN6* might be *APO1*. In substitution mapping of *qDPN6*, we found no significant differences in DPN among F_4_ lines (Fig. 2). However, DPN tended to be lower in F_4_ lines with low VBN, may be because DPN depends more on environmental conditions whereas VBN depends more on genetic control. Takeoka (1977) mentioned that the histogenesis of the inner VBN or VBN linked to the development of young panicles. Therefore, the gene on this location mainly controlled VBN as the histogenesis of the VBN at development of young panicles. Subsequently, the histogenesis of the VBN might influence to the size of DPN. Therefore, VBN could be clearly characterized in the F_4_ and F_5_ lines, while DPN was unclear. It will be necessary to confirm effects of *qDPN6* as future study.

We verified each detected QTL for DPN and VBN on its respective chromosome in CSSLs with the ‘IR24’ genetic background. *qDPN5* and *qDPN11* had a strong effect on DPN but a weak effect on VBN; *qDPN6* had a weak effect on DPN but a strong effect on VBN (Tables 2, 3, Supplemental Fig. 3). To examine QTL interactions associated with DPN, we used PYLs carrying pairs of QTLs. PYL4 (*qDPN5* + *qDPN6* + *qDPN11*) had reduced DPN, similar to that of ‘Asominori’ (Table 3). This result implies additive effects among QTLs. It was similar to the additive effect on VBN between *qVBN6* and *qVBN11* (Nguyen *et al.* 2023). A common positive correlation between DPN and VBN is associated with the capacity to transport matter from leaf to stem (Lee *et al.* 1992, Liao *et al.* 2021, Liu *et al.* 2008, Xu *et al.* 2005). VBN is significantly positively correlated with panicle structure, such as primary branch number, secondary branch number, spikelet number, and yield-related traits (Fukuyama and Takayama 1995, Liao *et al.* 2021, Nguyen *et al.* 2023, Sasahara *et al.* 1999, Zhai *et al.* 2018). We also found a correlation between DPN and VBN in the RILs (Supplemental Fig. 5) and F_2_ populations. The results of our study using the ‘IR24’ genetic background suggest that combining QTLs for DPN can reduce DPN and VBN and the reduction of VBN would be reduced translocation, the filled spikelets on the panicle, and yield potential.

In previous study, Nguyen *et al.* (2023) found three stable QTLs for VBN, *qVBN5*, *qVBN6* and *qVBN11* on RILs of the cross between ‘Asominori’ and ‘IR24’. These QTLs for VBN were evaluated the effects under ‘IR24’ and ‘Asomiori’ genetic backgrounds. To confirm the relationships among VBN and yield-related traits under *japonica* genetic background, VBN and yield-related traits of three CSSLs carrying ‘IR24’ alleles in the ‘Asominori’ genetic background (AIS38, AIS49, and AIS76) were observed (Nguyen *et al.* 2023). Spikelet number per panicle and VBN of AIS49 (carrying ‘IR24’ allele at *qDPN6*) and AIS76 (carrying ‘IR24’ allele at *qDPN11*) were increased relative to ‘Asominori’. These results correspond to the effects of *APO1* in a previous study: the ‘Habataki’ allele of *APO1* increased primary branch number and VBN, increasing grain yield per plant by enhancing spikelet number and carbohydrate translocation to panicles (Terao *et al.* 2010). Additionally, DPN of AIS38 (carrying ‘IR24’ allele at *qDPN5*), AIS49, and AIS76 in the ‘Asominori’ genetic background slightly increases but no significant difference from ‘Asominori’ (data not shown). These QTLs had tendency of increasing VBN, DPN and spikelet number of CSSLs with the ‘Asominori’ genetic background. The QTLs for VBN plus DPN on Chrs. 5 and 6 could be introduced into other *japonica* cultivars and used in developing *indica*–*japonica* cross cultivars and higher-yielding cultivars to improve yields through marker-assisted breeding.

## Author Contribution Statement

All authors contributed to the study conception and design. HTLN and YA prepared the material, collected data, and performed analyses. HTLN, SHZ, and DF wrote the manuscript.

## Supplementary Material

Supplemental Figures

Supplemental Tables

## Figures and Tables

**Table 1.** Detection of QTLs for diameter of panicle neck in RILs

QTL	Chr.	Marker interval	Physical distance (Mbp)*^a^*	LOD	Additive effect*^b^*	*R*^2^ (%)
*qDPN5*	5	RM18897–RM18910	23.88–24.13	6.3	–0.11	17.3
*qDPN6*	6	C962–Ky11	28.64	9.5	–0.17	38.1
*qDPN11*	11	Xnpb189-1–C718	2.03–1.89	4.7	–0.10	11.7

*^a^* On ‘Nipponbare’ genome sequence. *^b^* Effect of ‘Asominori’ allele.

**Table 2.** Validation of effect of a single QTL for diameter of panicle neck in CSSLs with ‘IR24’ genetic background

Line	QTL	Diameter of panicle neck (mm)		Vascular bundle number
		2020	2021	2021
IR24		2.74 ± 0.17	2.57 ± 0.12	22.2 ± 2.4
IAS30	*qDPN5*	2.28 ± 0.10**	2.01 ± 0.06***	20.6 ± 4.4
IAS39	*qDPN6*	2.45 ± 0.21*	2.38 ± 0.10*	15.8 ± 0.8**
IAS14	*qDPN11*	2.33 ± 0.19**	2.19 ± 0.05***	17.0 ± 2.8*

Asterisks: *P*-values at *5%, **1%, and ***0.1% by Dunnett’s test.

**Table 3.** Effects of diameter of the panicle neck in pyramided lines with ‘IR24’ genetic background in 2020, 2021 and 2022

Line	QTL	Diameter of the panicle neck (mm) (Mean value ± SD)		
		2020	2021	2022
IAS30	*qDPN5*	2.23 ± 0.11^bc^	1.94 ± 0.15^ab^	1.97 ± 0.15^bcd^
IAS39	*qDPN6*	2.37 ± 0.15^c^	2.38 ± 0.11^c^	2.18 ± 0.19^de^
IAS14	*qDPN11*	2.25 ± 0.07^bc^	2.13 ± 0.07^b^	2.06 ± 0.09^cde^
PYL1	*qDPN5+11*	2.02 ± 0.16^ab^	1.89 ± 0.13^ab^	1.87 ± 0.11^bc^
PYL2	*qDPN6+11*	2.26 ± 0.13^bc^	2.04 ± 0.14^b^	1.83 ± 0.09^abc^
PYL3	*qDPN5+6*	2.06 ± 0.11^ab^	1.98 ± 0.06^ab^	1.98 ± 0.09^bcd^
PYL4	*qDPN5+6+11*	–	–	1.79 ± 0.14^ab^
Asominori		1.81 ± 0.09^a^	1.78 ± 0.17^a^	1.61 ± 0.13^a^
IR24		2.74 ± 0.17^d^	2.57 ± 0.12^c^	2.23 ± 0.12^e^

Values with the same letter are not significantly different between genotypes in each year 2020, 2021 and 2022 by Tukey–Kramer multiple comparison test (*P* < 0.05) in 2020, 2021 and 2022.

**Table 4.** Confirmation of QTLs for diameter of panicle neck in F_2_ populations derived from CSSLs

Population	QTL	Chr.	Marker interval	Physical distance (Mbp)*^a^*	LOD	Additive effect*^b^*	Dominant effect	*R*^2^ (%)
F_2_ (IAS30/​IR24)	*qDPN5*	5	RM7081–RM7446	24.59–25.02	5.5	–0.12	–0.04	15.9
F_2_ (IAS39/​IR24)	*qDPN6*	6	RM6395–RM20546	26.00–27.40	13.4	–0.18	–0.06	33.7

*^a^* On ‘Nipponbare’ genome sequence. *^b^* Effect of ‘Asominori’ allele.
