# Supplementary material for: Characterization of QTLs for diameter in panicle neck and substitution mapping of qDPN5/qVBN5.2 and qVBN6 in rice (Oryza sativa L.)
Source: Breed Sci. 2024 Aug 14;74(4):337–43. doi: 10.1270/jsbbs.23076 (PMC11769591; doi:10.1270/jsbbs.23076)
Supplement: Supplementary file 1 — Supplemental Figures [file 74_337-s1.pdf]

doi: 10.1270/jsbbs.23076

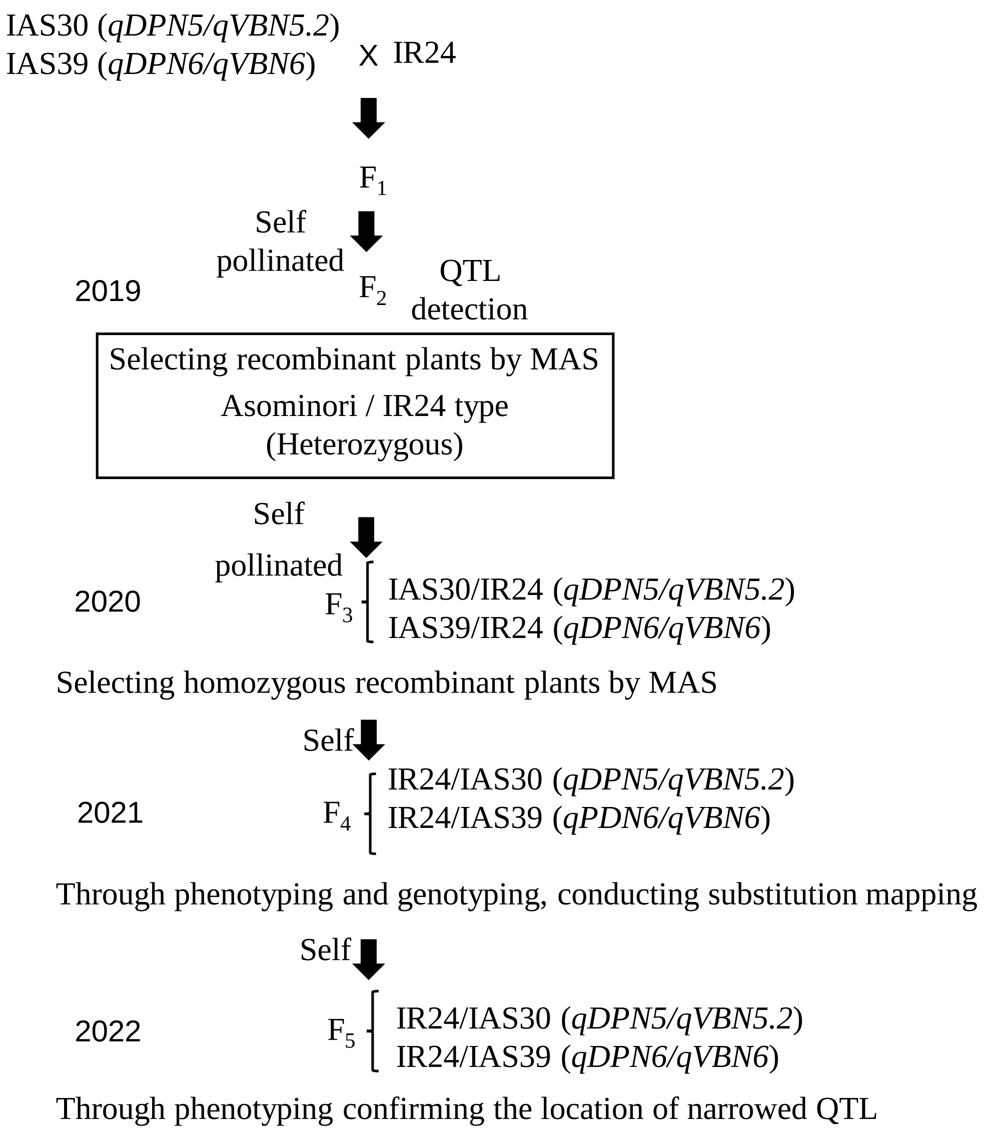

Supplemental Fig. 1. Breeding scheme for substitution mapping

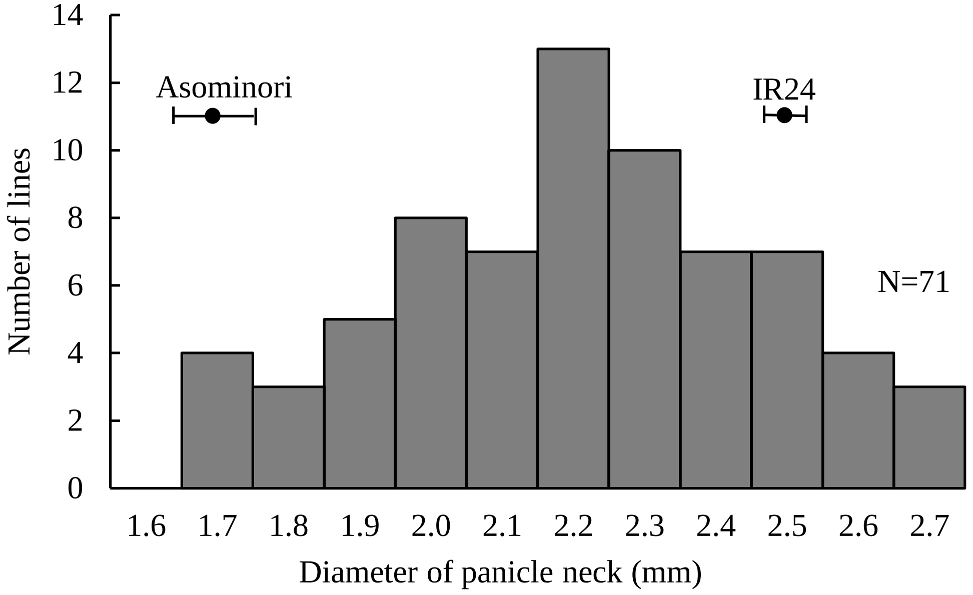

Supplemental Fig. 2. Frequency distributions of diameter of panicle neck in RILs derived from a cross between *japonica* rice variety ‘Asominori’ and *indica* rice variety ‘IR24’.

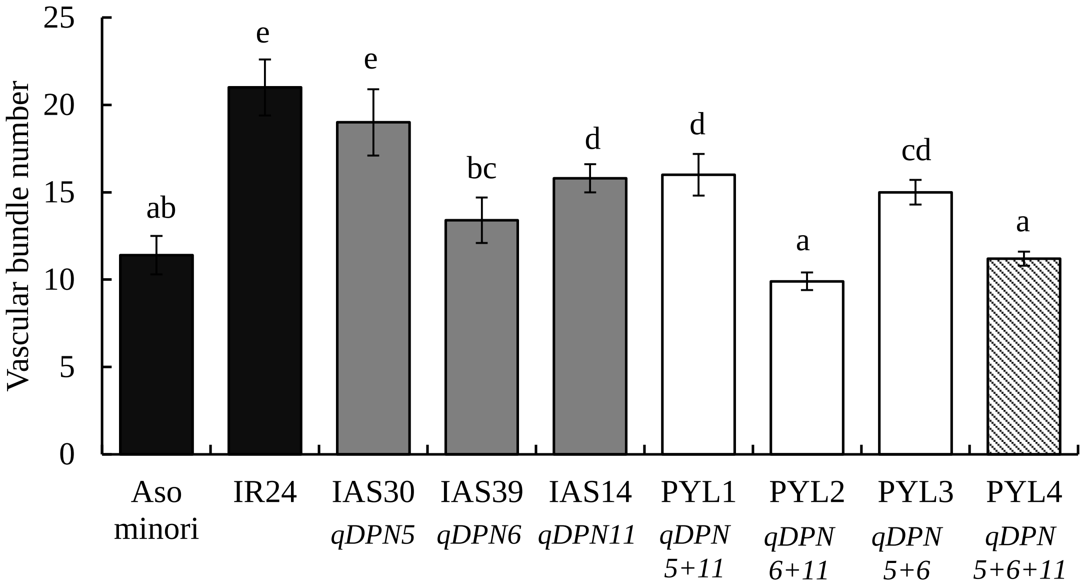

Supplemental Fig. 3. Effects of pyramiding of 3 QTLs for vascular bundle number in 'IR24' genetic background. Bars with the same letter are not significantly different between genotypes by Tukey–Kramer multiple comparison test ($P < 0.05$).

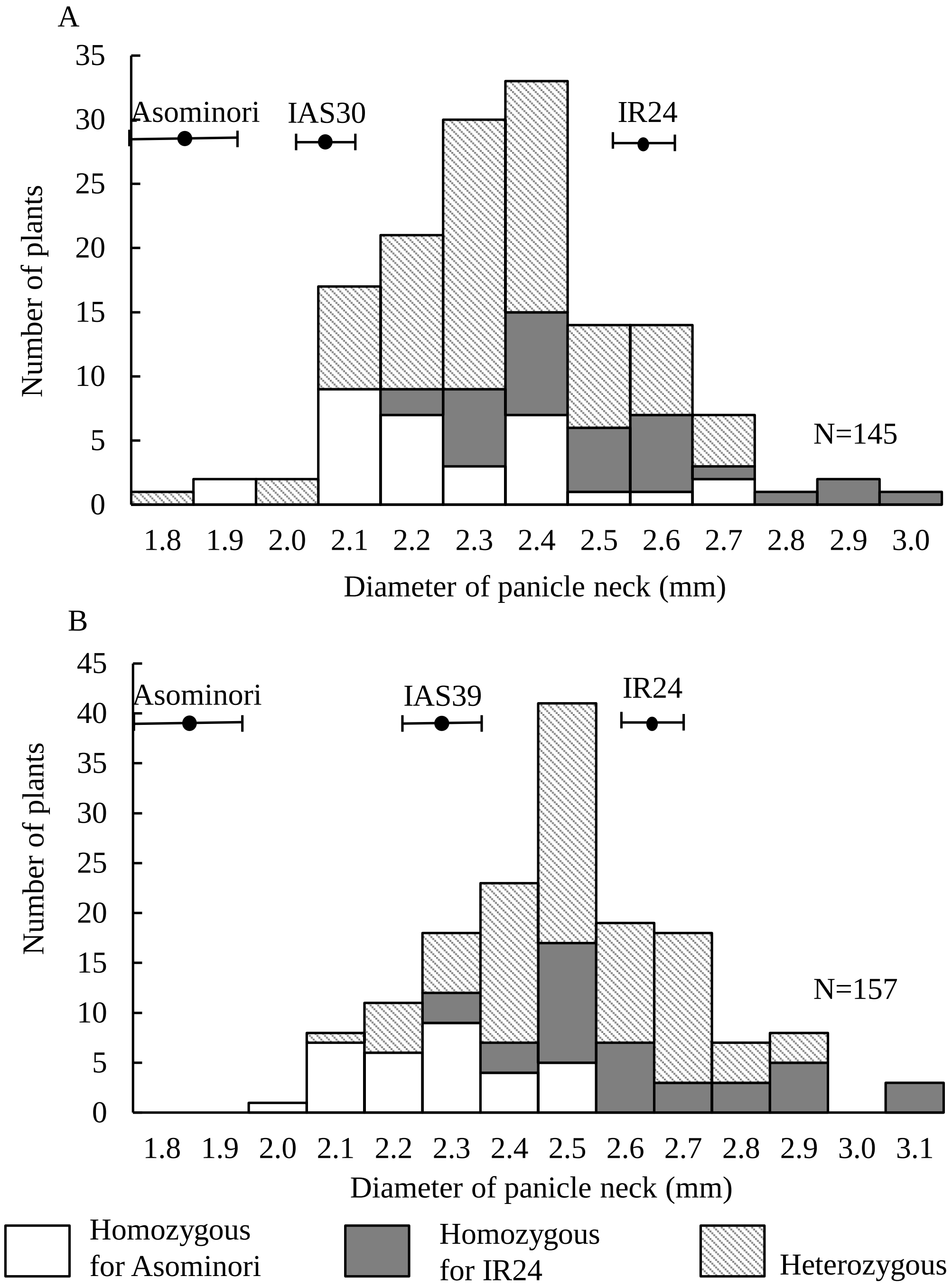

Supplemental Fig. 4. Frequency distributions of diameter in panicle neck in (A) F_2 population from IAS30/IR24 (B) F_2 population from IAS39/IR24. The genotype of RM7081 in F_2 of IAS30/IR24 and RM20546 in F_2 of IAS39/IR24 were classified into the three groups with different colors (white bar: homozygous for 'Asominori', black bar: homozygous for 'IR24', and shaded bar: heterozygous). Bars indicate means in parents with standard deviation.

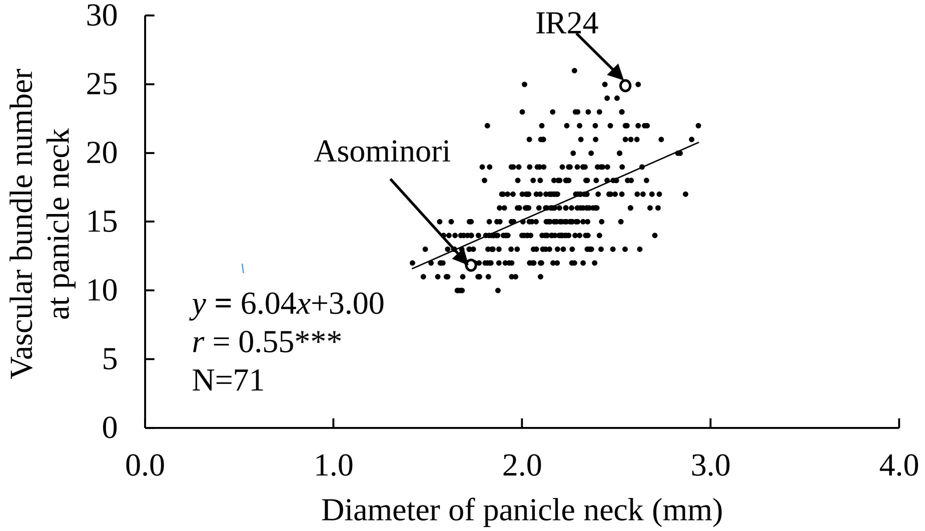

Supplemental Fig. 5. Relationship between diameter of panicle neck and vascular bundle number at panicle neck in 71 RILs that were derived from a cross between 'Asominori' and 'IR24'. The regression lines and correlation coefficient are indicated.
