# Supplementary material for: Characterization of QTLs for diameter in panicle neck and substitution mapping of qDPN5/qVBN5.2 and qVBN6 in rice (Oryza sativa L.)
Source: Breed Sci. 2024 Aug 14;74(4):337–43. doi: 10.1270/jsbbs.23076 (PMC11769591; doi:10.1270/jsbbs.23076)
Supplement: Supplementary file 2 — Supplemental Tables [file 74_337-s2.pdf]

doi: 10.1270/jsbbs.23076

Supplemental Table 1. The SSR and indel markers for maker-assisted selection of three QTLs on Chrs. 5, 6 and 11

Marker[a]	Chr.	Forward primer sequence (5' -> 3')	Reverse primer sequence (5' -> 3')	Physical position (Mbp)[b]	Predicted size (bp)[c]
RM3351	5	GTCGAAACGTAGCCAGGCAATGG	CCATGGAAGGAATGGAGGTGAGG	20.62	139
RM6841	5	CTTCCCGAAATCAGATTCTTGC	CGACGAGTCCTACACACTCTCC	22.66	576
RM6395	6	GGCTTCGGCTTCTGAACTAGC	CGACTAAGCAGCAGTAACAATCTCG	25.61	93
RM400	6	TTACACCAGGCTACCCAAACTCG	TTGCTGAGTTCCCTCGTCTATCC	28.05	380
C5-indel8795	11	GAACCAATGTTGAGATGTTTCC	GAAAGCAATTTCGCTTATCTCAA	0.86	139
C5-indel8837	11	GAACATGCAAGGACCATACTAAA	GGGTAGTGCTTGTTTATTTCAA	1.83	150

[a] Markers with RM were from McCouch *et al.* 2002, while C5- indel were from Yonemaru *et al.* 2015

[b] The marker's physical position was determined by the forward primer's location, obtained from The Rice Annotation Project Database

[c] The predicted size is determined by the reference genome ('Nipponbare' IRGSP v.1.0).

Supplemental Table 2. The markers for maker-assisted selection of chromosomes 5 and 6

Marker[a]	Chr.	Forward primer sequence (5' -> 3')	Reverse primer sequence (5' -> 3')	Physical position (Mbp)[b]	Predicted size (bp)[c]
RM6054	5	AGGCTCTTCGGCTTCATCTCC	GGTCTCTGATCAGTTTGCTTTGG	22.70	190
KNJ8 indel384	5	ACGTCAGCGCCTGATTTC	TATTCTGTGTTCGAGCCATGTG	23.47	145
KNJ8 indel385	5	GCCACGTGGACTATAGAGGAGA	CCCAAGTCAATCCAATAGTTTCTT	23.52	142
RM18910	5	CACCCAATATGAGTACGGAACAGC	ATACTTGCCGGTATCTTCGTTCC	23.98	153
RM18914	5	GCTCTCCATGTGTTATCAGCAACC	GAGAAAGAGTTTCTTCTGCCTGTTGG	24.04	299
RM18926	5	CTACCTCTCCGTCCGCATCC	AAGAGGGACTCAGAGAGCACTTCC	24.34	249
RM7081	5	CTTCCCGCACTACACTGCACTCC	CTGCAACTTGCTCATGGAGTTGG	24.59	97
RM7446	5	CGTTGAGCCAAGAAGAAGAAAGG	TTTGAAGGCAGTTTCACTGACG	25.02	195
RM3348	5	CTTCTCGGTTCATCCAAAGAGC	GTGGAAGCTATGGGTAGCTCACG	24.95	88
C5 Indel4925	5	CGCTCTGGATTTATCAGCTTCT	TATGGGTAGCTCACGTGGAGAG	25.15	97
C5 Indel 5718	6	CCACTTAGGAATAAAGGCTGGA	TCTATCCGGTCTTGAAACGAGT	25.84	136
C5 Indel 5740	6	CACGATTGATGATTTCACTTGC	ACGCTCTCCCGCCACAAC	26.38	117
C5 Indel 5755	6	CCGTTGGTTCTCTCTACTCACC	AAGCCCATGTCCTTATCCCTAA	26.83	135
C5 Indel 5756	6	GCCAAAGATCCATAGTTCAACC	CATGGATCCACCTGTATGAATC	26.90	112
RM20546	6	TGAGCAGGAGACGGGACAGC	TATCCGTTTCTGCAACGCTACGC	27.40	170
KNJ8 Indel493	6	TTTCTGCTCCTGAAACACGTTA	GCAGCACAAAGCTCTATCTATCA	27.65	149
RM20596	6	AACTTCCTTTCCAGGCTTTCAGC	TTCACTGAGCCTGAACACATTGC	28.09	170
RM400	6	TTACACCAGGCTACCCAAACTCG	TTGCTGAGTTCCCTCGTCTATCC	28.43	380
RM 3138	6	GTGGTGAATGTTGAGCTGCATGG	GACTGAGCCAAGTTGCTGTCTGG	28.47	199

[a] Markers with RM were from McCouch *et al.* 2002, while KNJ8- and C5-indel were from Yonemaru *et al.* 2015

[b] The marker's physical position was determined by the forward primer's location, obtained from The Rice Annotation Project Database

[c] The predicted size is determined by the reference genome ('Nipponbare' IRGSP v.1).
